# Structural and Luminescence Behavior of Nanocrystalline Orthophosphate K*Me*Y(PO_4_)_2_: Eu^3+^ (*Me* = Ca, Sr) Synthesized by Hydrothermal Method

**DOI:** 10.3390/ma15051850

**Published:** 2022-03-01

**Authors:** Aleksandra J. Pelczarska, Dagmara Stefańska, Adam Watras, Lucyna Macalik, Irena Szczygieł, Jerzy Hanuza

**Affiliations:** 1 Department of Inorganic Chemistry, Faculty of Production Engineering, Wroclaw University of Economics and Business, Komandorska Street 118/120, 53-345 Wroclaw, Poland; irena.szczygiel@ue.wroc.pl; 2 Institute of Low Temperature and Structure Research, Polish Academy of Science, Okolna Street 2, 50-422 Wroclaw, Poland; a.watras@intibs.pl (A.W.); l.macalik@intibs.pl (L.M.); j.hanuza@intibs.pl (J.H.)

**Keywords:** europium doped phosphate, nanocrystallites, hydrothermal synthesis, optical properties, IR, Raman, emission studies

## Abstract

K*Me*Y(PO_4_)_2_:5% Eu^3+^ phosphates have been synthesized by a novel hydrothermal method. Spectroscopic, structural, and morphological properties of the obtained samples were investigated by X-ray, TEM, Raman, infrared, absorption, and luminescence studies. The microscopic analysis of the obtained samples showed that the mean diameter of synthesized crystals was about 15 nm. The KCaY(PO_4_)_2_ and KSrY(PO_4_)_2_ compounds were isostructural and they crystallized in a rhabdophane-type hexagonal structure with the unit-cell parameters *a* = *b* ≈ 6.90 Å, *c* ≈ 6.34 Å, and *a* = *b* ≈ 7.00 Å, *c* ≈ 6.42 Å for the Ca and Sr compound, respectively. Spectroscopic investigations showed intense ^5^D_0_ → ^7^F_4_ transitions connected with D_2_ site symmetry of Eu^3+^ ions. Furthermore, for the sample annealed at 500 °C, europium ions were located in two optical sites, on the surface of grains and in the bulk. Thermal treatment of powders at high temperature provided better grain crystallinity and only one position of dopant in the crystalline structure. The most intense emission was possessed by the KSrY(PO_4_)_2_:5% Eu^3+^ sample calcinated at 500 °C.

## 1. Introduction

In recent times, inorganic phosphors doped with lanthanide ions have attracted much attention due to their potential applications as phosphors, laser materials, or solid-state lighting [1,2,3,4,5,6]. The current white-light-emitting diodes (W-LEDs) suffer from a lack of red light, therefore, red phosphors are sought to fill deficiency. Generally, W-LEDs are fabricated by combining the yellow emission of YAG: Ce^3+^ with the blue light of the diode [7]. The second method is to mix the blue, green, and red emissions generated from three or two blended powders. Rare-earth-doped (*RE*) phosphates are a good candidate for the formation of white LEDs due to their good physical and chemical stability, low cost of substrates, and reduced preparation temperatures compared to silicates, aluminosilicates, or nitrates [8,9,10,11,12,13].

**Figure 1:**
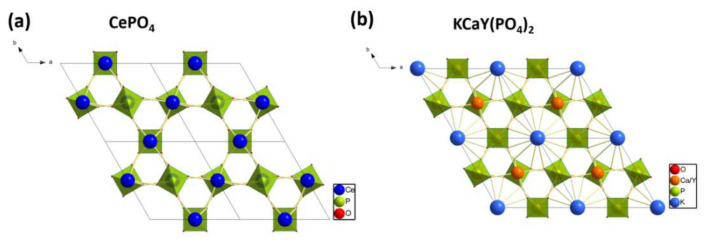
(**a**) A visualization of the rhabdophane type of structure of CePO_4_, and (**b**) of the KCaY(PO_4_)_2_ structure.

One of the most promising phosphors are K*MeRE*(PO_4_)_2_ phosphates, where *Me* = Ca or Sr, and have a hexagonal structure with the unit cell parameters *a* = b ≈ 7.0 and *c* ≈ 6.4 Å [14,15,16,17,18]. The structure is closely related to rhabdophane (*RE*PO_4_·*n*H_2_O), in which half of the cationic sites of *RE*^3+^ are filled by *Me*^2+^ (Figure 1). Simultaneously, the electrostatic charge is compensated by the placement of K^+^ in free spaces inside of channels running parallel to the *c* axis and formed by (*RE*, Ca)-PO_4_-(*RE*, Ca) chains [14]. According to the available literature, the simultaneous replacement of *RE*^3+^ with K^+^ and Ca^2+^ reduces the group symmetry of PO_4_ and *R*O_8_ (*R* = Ca or *RE*) from D_2_ to C_2_ [17].

The K*MeRE*(PO_4_)_2_ phosphates show good luminescence and hence can be applied as efficient luminophores [19,20]. Recently, a series of K*MeRE*(PO_4_)_2_ (*Me* = Ca, Sr; *RE* = Y, La, Lu, Gd) have been investigated carefully [4,21,22,23,24]. Wang et al. successfully synthesized KCaY(PO_4_)_2_:Dy^3+^, Eu^3+^ as a nanosized phosphor-generated warm-white light with a high color rendering index (CRI) [23]. Basic information about KCaY(PO_4_)_2_:Eu^3+^ has been reported in 2006 by Wang et al. [24]. Furthermore, the energy transfer from Eu^2+^ to Mn^2+^ in KCaY(PO_4_)_2_ was studied by Liu and co-workers [4]. Up until now, strontium ternary phosphates were activated by *RE* ions, such as Ce^3+^, Tb^3+^, Eu^3+^, and Mn^2+^ [21,22]. Samples doped with Ce^3+^, Tb^3+^, and Mn^2+^ exhibited high quantum efficiency (60%) and were obtained by a Pechini method [22]. Compounds are mostly obtained via a conventional ceramic method (sintering at high temperatures), however, results presented in [25,26,27] show that obtaining single-phase K*MeRE*(PO_4_)_2_ is difficult because of the limited thermal stability of such ternary phosphates at temperatures exceeding 1100–1200 °C. A relatively mild hydrothermal process (short-term heating carried out at 240 °C) has been applied for the synthesis of phase-pure KCaCe(PO_4_)_2_, which resulted in obtaining nanocrystalline powders [27]. 

The aim of the presented study was to describe the structural and luminescence properties of nanocrystalline K*Me*Y(PO_4_)_2_ (*Me* = Ca, Sr) powders doped with Eu^3+^ and to discuss the obtained results from the point of view of the powders’ morphology. 

## 2. Materials and Methods

The powders of K*Me*Y(PO_4_)_2_:5% Eu^3+^ (*Me* = Ca or Sr) were obtained by a short-lasting hydrothermal technique. In the first step, Y_2_O_3_ (99.99% POCh, Gliwice, Poland) and Eu_2_O_3_ (99.9%, POCh, Gliwice, Poland) were digested in dilute nitric acid. The obtained clear solutions were dried for several days over solid NaOH until solid nitrates crystallized. The starting sols for hydrothermal processes were prepared as follows. Rare earth nitrates and Ca(NO_3_)_2_·4H_2_O (≥99.0%, POCh, Gliwice, Poland) or Sr(NO_3_)_2_ (≥99.0%, Sigma-Aldrich, Steinheim, Germany) were dissolved in 10 cm^3^ of distilled water, then K_3_PO_4_·H_2_O (≥94%, Fluka, Steinheim, Germany) was added to the solution, and the obtained mixture was diluted to 50 cm^3^ volume. The molar ratio of K:*Me*:Y:Eu metals in the obtained sols was 68:1:0.95:0.05. After one-day ageing, the sols were used in a hydrothermal process performed in a Magnum II autoclave (Ertec, Wrocław, Poland) with heating of the sols by microwaves of 600 W maximal power. The syntheses were controlled by temperature measurements performed at a bottom part of the reactor made of PTFE. The syntheses were carried out for 2 h at a maximal temperature of 250 °C and a pressure of 30 bar. As they were obtained, fine precipitates were centrifuged, washed several times with distilled water, and dried for 24 h at 120 °C. Further heating of the samples was carried out at 500 °C and 1000 °C for 10 h. 

Phase analysis of obtained powders was performed using the XRD technique and a Siemens D5000 diffractometer (Munich, Germany) equipped with a copper radiation cathode. The measurements were performed at a 2θ angle range of 5–60° with a 0.02° step and at least 4 s per step. The indexing of powder patterns was made using the computer program DICVOL [28]. Unit cell lattice constants of K*Me*Y(PO_4_)_2_:5%Eu were refined using the Checkcell software (LMGP, Grenoble, France) [29].

Simultaneous TGA/DSC measurements were carried out using a SETSYS^TM^ (TG-DSC 1500; Setaram, Geneva, Switzerland) in the temperature range of 20–1200 °C (sample mass 40 mg, platinum crucibles, heating rate 10 °C min^−1^, argon atmosphere).

Excitation spectra were measured on a McPherson spectrometer (McPherson Inc. Chelmsford, MA, USA) equipped with a 150 W Xe lamp and 150 W deuterium lamp (for VUV part) as excitation sources, and a Hamamatsu R928 photomultiplier (Hamamatsu Photonics, Hamamatsu, Japan) as a detector. The normalization of the XRD and excitation spectra was performed at the highest point of the spectrum. The emission spectra were measured using a Hamamatsu PMA12 multichannel analyzer (Hamamatsu Photonics, Hamamatsu, Japan) as a detector, and 395 nm line from tunable pulsed Ti: Sapphire laser pumped by second harmonic (λ = 532 nm) of Nd: YAG laser as an excitation source (Lotis TII, Minsk, Belarus). Decay time measurements were performed using the same excitation as for emission spectra, while decay curves were collected on a Tektronix MSO-3052 digital oscilloscope (Tektronix, Beaverton, OR, USA). The low temperature measurement was carried out at 77 K using a quartz dewar. 

## 3. Results and Discussion

### 3.1. Structure and Morphology

**Figure 2:**
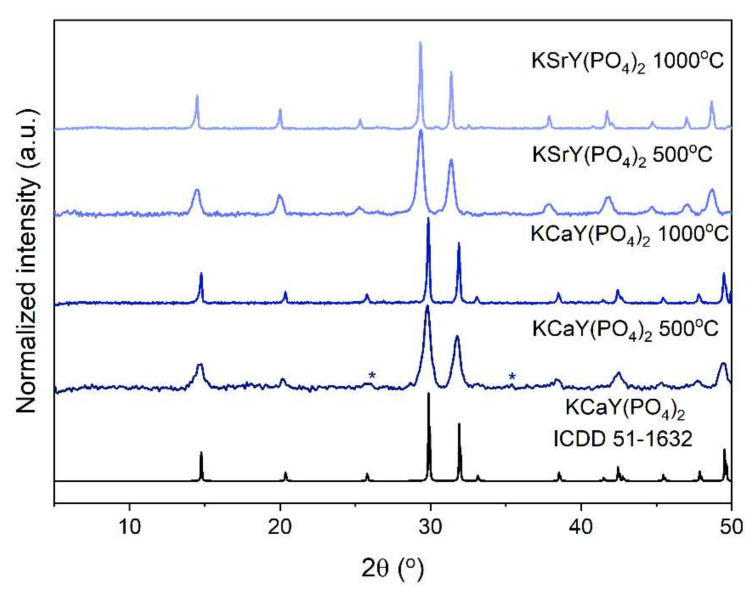
The XRD patterns of samples sintered at different temperatures. Asterisks denote unidentified reflections.

The analysis of XRD patterns of obtained phosphates confirmed the crystalline characteristics of the powders. All of the samples were isostructural to the rhabdophane-type KCaY(PO_4_)_2_ phosphate (ICDD card No. 51-1632) (Figure 2). The XRD pattern of dried samples showed pure structures after the hydrothermal process (Figure S1). Due to the presence of hydroxyl groups originating from zeolitic water molecules, which can easily quench luminescence, samples were annealed at 500 °C and 1000 °C. The annealing process does not change the structure of prepared phosphates. Minor reflections of a secondary phase were registered in the XRD pattern of KCaY(PO_4_)_2_:5%Eu^3+^ sintered at 500 °C. They most likely derive from the YPO_4_ xenotime phase; however, due to the low intensity of reflections, the presence of the phase can be not confirmed with reliability. The refined hexagonal unit cell parameters of the phosphates sintered at 1000 °C are listed in Table 1. 

**Figure 3:**
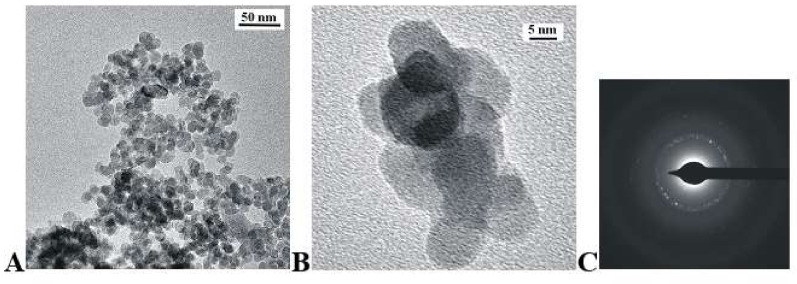
TEM (**A**,**B**) and SAED (**C**) images of KCaY(PO_4_)_2_:5% Eu^3+^ obtained by drying hydrothermally obtained powders.

**Figure 4:**
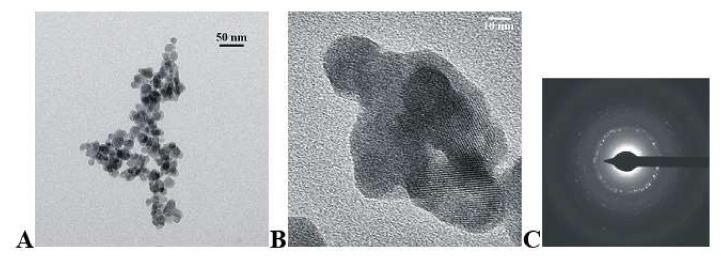
TEM (**A**,**B**) and SAED (**C**) images of KSrY(PO_4_)_2_:5% Eu^3+^ obtained by drying of hydrothermally obtained powders.

Figure 3 and Figure 4 present the TEM and SEAD representative images of KCaY(PO_4_)_2_:5% Eu^3+^ and KSrY(PO_4_)_2_:5% Eu^3+^ samples, respectively. As can be seen, the obtained compounds possess rounded grains with an average particle length size (maximum particle dimension) D[1, 0] equal to 12 ± 2 nm and 18 ± 4 nm for Ca and Sr phosphates, respectively. The powders consist of single grains, as well as bigger objects created by the clumping of a few grains. Agglomerates have an irregular shape made from connected spherical single nanocrystals. The replacement of calcium ions by the strontium one does not change the shape of obtained nanopowders.

**Figure 5:**
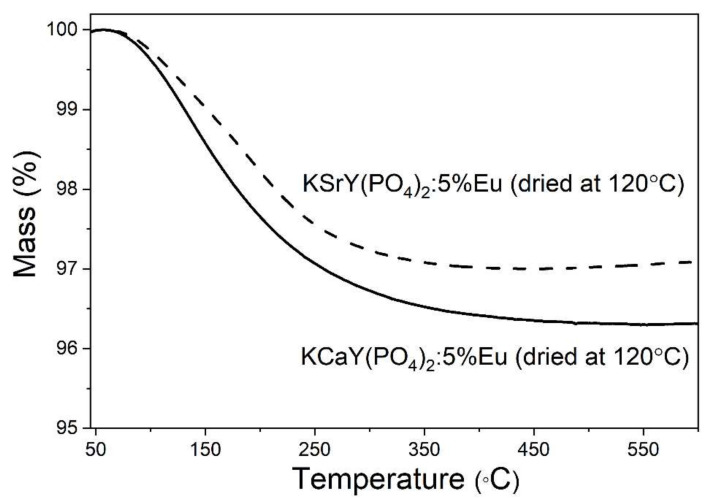
The TGA heating curves of samples obtained hydrothermally and dried at 120 °C.

The analysis of TGA heating curves of samples obtained hydrothermally showed a 3–4% mass loss of powders in the temperature range of 100–350 °C (Figure 5). Mass loss is characteristic of hydrated samples. A relatively high temperature of the effect suggests the presence of zeolitic-bound water. No other thermal effects were observed on TGA/DSC heating curves.

### 3.2. Raman and IR Studies

**Figure 6:**
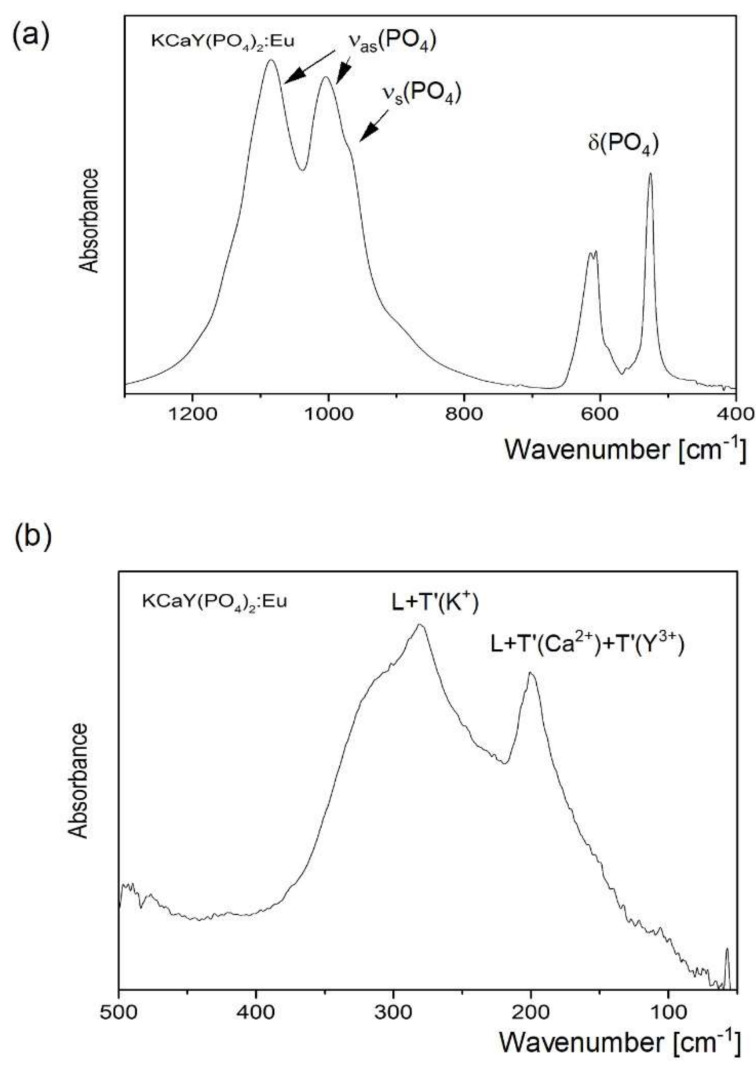
Infrared absorption spectra of KCaY(PO_4_)_2_:Eu^3+^ in the (**a**) FIR and (**b**) MIR range. Position of the stretching (ν) and bending (δ) modes as well as libration and translation are marked in the figures.

**Figure 7:**
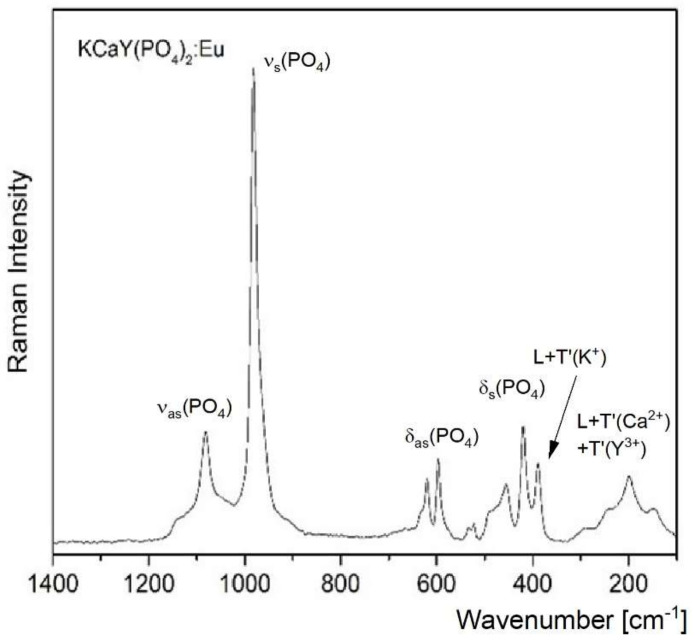
Raman spectra of KCaY(PO_4_)_2_:5% Eu^3+^.

Figure 6a,b present infrared spectra in the middle (MIR) and far (FIR) infrared range, and Figure 7 shows the Raman spectra. The spectra measured for the strontium derivative are identical, so we decided to only show spectra for the calcium compound as the representative. They were also similar to those reported earlier by Tie et al. [17]. In general, the bands observed in these spectra agree with those reported earlier for other orthophosphates [30,31,32,33]. The particular types of vibrations appear in the typical range. The ν_3_(F_2_) = ν_as_(PO_4_) vibrations were observed at 1130 and 1090 cm^−1^, where a strong multiplet consisting of 2–3 bands appeared. In the range characteristic for ν_1_(A_1_) = ν_s_(PO_4_) vibrations, a strong single Raman band was observed. All bending δ(PO_4_) vibrations were observed in the range of 350–650 cm^−1^, in which nine Raman and five IR bands could be identified. The IR bands of average to strong intensities in the ranges of 250–350 cm^−1^ and 150–250 cm^−1^ correspond to the T’(K^+^) and T’(Ca^2+^) translations, respectively. The analysis of these vibrations was performed in Reference 4 on the basis of the D_2_ site symmetry of this unit, although factor group splitting was not taken into account.

According to the above-cited references [14,15,16,17,18], K*Me*Y(PO_4_)_2_ phosphates (*Me* = Ca, Sr) crystallize in the hexagonal P6_2_22 (D_6_^4^) structure with Z = 3. Since the occupation of Ca^2+^, Nd^3+^, and K^+^ cations was equal to 0.5, a proper chemical formula can be written as (Ca_1.5_Y_1.5_)K_1.5_(PO_4_)_3_ with Z = 1 [14].

Its primitive unit cell contains three Ca^2+^/Y^3+^ ions equally sharing the 3c (D_2_ site) position, and three PO_4_^3−^ ions with P and O atoms in the 3d (D_2_) and 12k (C_1_) positions, respectively. The K^+^ ions could not be used for calculations because their occupancy in the primitive cell was partial. For this reason, only a partial factor group analysis can be presented.

The three PO_4_^3−^ ions and the Ca^2+^/Y^3+^ ions in the primitive cell give rise to 54 zone–center degrees of freedom described by the irreducible representation: Γ_v_ = 3A_1_ + 5A_2_ + 5B_1_ + 5B_2_ + 10E_1_ + 8E_2_ (54). Three of them, Γ_T_ = A_2_ + E_1_ (3), describe the acoustic phonons, and the remainder, Γ_O_ = 3A_1_ + 4A_2_ + 5B_1_ + 5B_2_ + 9E_1_ + 8E_2_ (51), correspond to the optical modes. The optical modes can be further subdivided by the vibrations of the respective components of the primitive cell, i.e., translations of Ca^2+^/Y^3+^ ions: Γ_T_(Ca^2+^/Y^3+^) = B_1_ + B_2_ + E_1_ + E_2_ (6), internal vibrations of PO_4_^3−^ ions: Γ_v_(PO_4_) = 3A_1_ + 2A_2_ + 2B_1_ + 2B_2_ + 4E_1_ + 5E_2_ (27), as well as translations and librations of the PO_4_^3−^ ions, Γ_T/L_(PO_4_) = A_2_ + B_1_ + B_2_ + 2E_1_ + E_2_ (6).

In the discussion of the IR and Raman spectra, the correlation between the molecular T_d_, site D_2_, and factor D_6_ symmetry should be used. The following schemes related to the correlation T_d_ → D_2_ → D_6_: for the ν_1_ mode A_1_ → A → A_1_ + E_2_; for the ν_2_ mode E → 2A → 2A_1_ + 2E_2_; and for the ν_3_ and ν_4_ modes F_2_ → B_1_ + B_2_ + B_3_ → A_2_ + B_1_ + B_2_ + 2E_1_ + E_2_. Since the A_2_ and E_1_ modes were IR-active, A_1_, E_1_, and E_2_ were Raman-active, and B_1_ and B_2_ were silent for *P*6_2_22 symmetry, at least 13 (4A_2_ + 9E_1_) IR bands were expected in this spectrum, and similarly, 20 (3A_1_ + 9E_1_ + 8E_2_) bands should be observed in the Raman spectrum. These data fit well to the number of bands observed in the IR and Raman spectra, i.e., 14 and 20, respectively (Figure 6 and Figure 7).

### 3.3. Optical Studies

**Figure 8:**
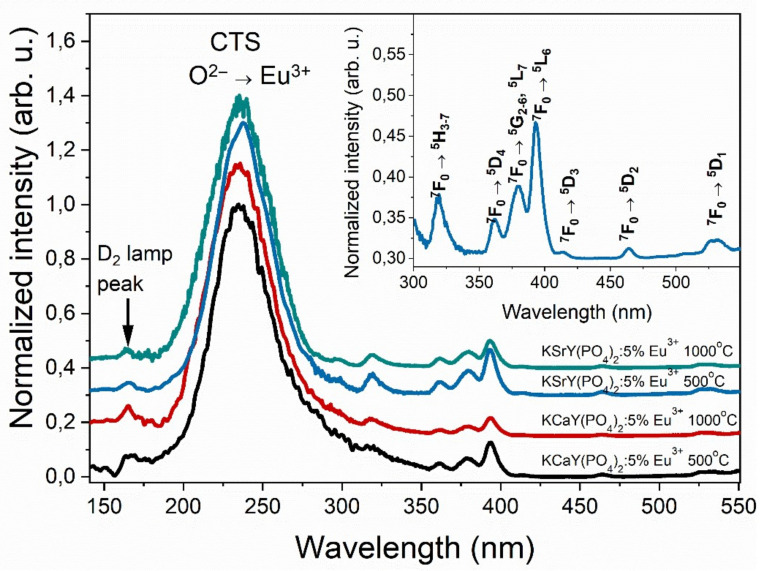
Excitation spectra of K*Me*Y(PO_4_)_2_:5% Eu^3+^ samples monitored at λ_em_ = 612 nm, and the assigned f–f absorption bands of representative KSrY(PO_4_)_2_:5% Eu^3+^ 500 °C sample (inset).

The excitation spectra of K*Me*Y(PO_4_)_2_:5% Eu^3+^ were measured by observing the transition at λ_em_ = 612 nm (Figure 8). The spectra consisted of a broad and intense band centered at 235 nm, which was assigned to the charge transfer transition from oxygen to europium ions (CTS—charge-transfer state) [34]. Besides the CTS, there were also sharp and narrow bands, which derived from the 4f–4f transition of Eu^3+^ [35]. The bands were assigned to the ^7^F_0_ → ^5^H_3–7_ at 319 nm, ^7^F_0_ → ^5^D_4_ at 361.5 nm, ^7^F_0_ → ^5^G_2–6_, ^5^L_7_ at 379 nm, ^7^F_0_ → ^5^L_6_ at 393 nm, ^7^F_0_ → ^5^D_3_ at 413.5 nm, ^7^F_0_ → ^5^D_2_ at 464 nm, and ^7^F_0_ → ^5^D_1_ at 532 nm. All spectra were normalized to the intensity of the charge transfer band. In the sample sintered at 1000 °C, the ratio between the CTS and 4f–4f bands increased.

**Figure 9:**
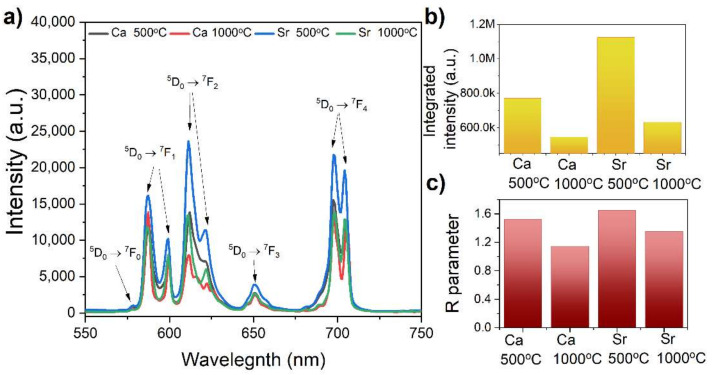
(**a**) The emission spectra of K*Me*Y(PO_4_)_2_:5% Eu^3+^, λ_exc_ = 393 nm, (**b**) the integrated intensity of registered emissions, and (**c**) the *R* parameter of investigated samples.

The room temperature emission spectra measured with excitation at λ = 393 nm consist of bands characteristic for Eu^3+^ ions’ emission (Figure 9a) [36]. They can be assigned to the transition from the ^5^D_0_ excited level to ^7^F_J_ (J = 0, 1, 2, 3, 4) ground levels. The emission peak attributed to the ^5^D_0_ → ^7^F_0_ was located with the maximum at 578 nm, while the ^7^F_1_ was at 587 nm, ^7^F_2_ was at 612 nm, and ^7^F_3_ and ^7^F_4_ were at 653 nm and 704 nm, respectively. The highest emission intensity was exhibited by the KSrY(PO_4_)_2_:5% Eu^3+^ sample annealed at 500 °C (Figure 9b). The Ca^2+^ analogous phosphate possessed lower emission intensity. Furthermore, heat treatment of samples at 1000 °C decreased the overall emission intensity for both KCaY(PO_4_)_2_:5% Eu^3+^ and KSrY(PO_4_)_2_:5% Eu^3+^. This was probably connected with more efficient non-radiative processes. When the samples were annealed at a higher temperature, all Eu^3+^ ions only occupied one position in the crystal structure of the investigated phosphate. As a consequence, the distance between Eu^3+^–Eu^3+^ ions decreased causing concentration quenching. The enhancement of non-radiative processes with annealing temperatures confirmed registered decay curves (see Figures S4–S7). As shown in Figure 9a, the most intense was the electric dipole transition (ED) ^5^D_0_ → ^7^F_2_. However, it should the emphasized that the intensity of ^7^F_4_ bands was anomalously intense. Similar behavior was reported for the first time in 1988 by Blasse [37]. He linked this situation with the D_4h_ site symmetry of Eu^3+^ ions. The same observation has been presented by Ferreira in Na_9_[EuW_10_O_36_]∙14H_2_O [38]. However, in the investigated phosphates, Eu^3+^ ions occupy Ca^2+^/Sr^2+^ sites with D_2_ symmetry. Bettinelli in 2011 explained that the distortion of EuO_8_ polyhedra to D_2_ symmetry was responsible for the enhancement of the ^5^D_0_ → ^7^F_4_ transition [39].

No significant changes were detected in the emission spectra recorded at 77 K (Figure S2). With the temperature decreasing, emission bands become narrower; however, even at low temperatures, the peaks overlapped and they were not well separated. The ^5^D_0_→^7^F_0_ transition of the sample annealed at 500 °C consisted of two peaks coming from two optical positions of dopant in the host of the compounds under study. On the other hand, for samples obtained at 1000 °C, only one ^7^F_0_ band was presented. The localization of Eu^3+^ ions on the surface of nanocrystals and inside the bulk can cause the occurrence of two dopant sites, and has been reported many times in the literature [40]. Presented emission and decay times also confirmed this hypothesis. The ^5^D_0_ → ^7^F_0_ transition of samples annealed at 500 °C consisted of two separate bands, while only one band was observed for samples prepared at 1000 °C. The magnification of the ^5^D_0_ → ^7^F_0_ transition of all samples is presented in Figure S3. These observations were in line with measured emission decay curves (see below). 

For europium ions, the magnetic dipole transition (^7^F_0_ → ^5^D_1_) was almost independent of the local symmetry and type of matrix, while the electric dipole transition (^7^F_0_ → ^5^D_2_) was very sensitive to any change in the local environment. When europium ions occupied a site with a center of symmetry, the only allowed transition was the magnetic dipole, while for europium ions at non-centrosymmetric sites, the most intense transition was the electric dipole. Therefore, the ratio between these transitions is called the asymmetry parameter *R* and is defined as follows:

R=∫D05→F27∫D05→F17

The higher the value of the *R* parameter, the more non-centrosymmetric the site of Eu^3+^ was. Calculated values of the *R* parameter are listed in Table 2 and Figure 9c. Increasing the annealing temperature of samples lowered the *R* parameter, which meant that distortion of the local surrounding of Eu^3+^ ions decreased, and for KCaY(PO_4_)_2_:5% Eu^3+^ annealed at 1000 °C, it was close to being ideally octahedral.

Room temperature decay curves measured for all samples are presented in Figures S4–S7, while the calculated decay times are listed in Table 2. The decay curves of samples annealed at 500 °C have biexponential characteristics, which confirmed the localization of europium ions on the nanograins surface and inside the bulk. The calculated values of decay time were typical for Eu^3+^ ions [35,41,42,43]; for example, the decay time of the Eu^3+^ ions in the Y_2_O_3_ compound equaled 2 ms, while for YAsO_4_:Eu^3+^ it was around 4 ms. Increasing the annealing temperature caused a decrease in decay time, which was in good agreement with the observed behavior of emission intensity. The enhancement of non-radiative processes with annealing temperatures confirmed registered decay curves. The lifetime of the KCaY(PO_4_)_2_:5% Eu^3+^ decreased from 5.4 ms to 4.7 ms with increasing annealing temperatures from 500 °C to 1000 °C, respectively.

## 4. Conclusions

Nanocrystalline powders of the K*Me*Y(PO_4_)_2_:5% Eu^3+^ (*Me* = Ca, Sr) composition were obtained at relatively mild hydrothermal conditions. The XRD study confirmed the crystal structure of the investigated ternary phosphates, and TEM images showed that irregular agglomerates consisted of particles with an average size below 20 nm. Careful study of the IR and Raman spectra allowed all the vibrations, translations, and librations to be assigned. K*(Ca, Sr)*Y(PO_4_)_2_ activated with 5% of Eu^3+^ ions showed intense red emission, with a dominance of electric dipole transitions from the ^5^D_0_ to ^7^F_2_ level. The local symmetry of Eu^3+^ ions in the investigated phosphates K*Me*Y(PO_4_)_2_:5% Eu^3+^ (*Me* = Ca, Sr) was D_2_, which caused the abnormally high intensity of the ^5^D_0_ → ^7^F_4_ transition. For samples annealed at the temperature of 500 °C, Eu^3+^ ions were on the surface of the nanocrystals and inside the bulk. Increasing the annealing temperature to 1000 °C resulted in better crystallinity of the sample and only one location of doping ions. Due to this, the distance between Eu^3+^–Eu^3+^ ions was reduced, which caused concentration quenching. For this reason, the most intense emission was observed for the samples heat-treated at 500 °C. These results were consistent with the recorded decay times.

## Figures and Tables

**Table 1:** The unit cell parameters and the cell volume of the K*Me*Y(PO_4_)_2_:5% Eu^3+^ powders sintered at 1000 °C.

Sample	Unit Cell Parameters (Å)		Cell Volume (Å^3^)
	*a = b*	*c*	
KCaY(PO_4_)_2_:5% Eu^3+^	6.8873 ± 0.0045	6.3380 ± 0.0004	260.4
KSrY(PO_4_)_2_:5% Eu^3+^	6.9911 ± 0.0015	6.4186 ± 0.0002	271.7

**Table 2:** The decay time and the *R* parameter for the K*Me*Y(PO_4_)_2_:5% Eu samples.

Sample	τ_1_ (ms)	τ_2_ (ms)	*R*
KCaY(PO_4_)_2_:5% Eu^3+^/500 °C	1.4	5.4	1.52
KCaY(PO_4_)_2_:5% Eu^3+^/1000 °C	4.7	---	1.14
KSrY(PO_4_)_2_:5% Eu^3+^/500 °C	1.7	4.6	1.65
KSrY(PO_4_)_2_:5% Eu^3+^/1000 °C	3.8	---	1.35

## Data Availability

Data are contained within the article.

## Supplementary Materials

The following supporting information can be downloaded at https://www.mdpi.com/article/10.3390/ma15051850/s1: Figure S1: XRD patterns of obtained samples after hydrothermal process without annealing; Figure S2: Emission spectra of representative samples K*Me*Y(PO_4_)_2_ (*Me* = Ca, Sr) recorded at 77 and 300 K; Figure S3: Magnification of the ^5^D_0_–^7^F_0_ transition of investigated compounds; Figures S4–S7: Emission decay curves of the K*Me*Y(PO_4_)_2_:5% Eu3+ (*Me* = Ca, Sr) samples recorded at room temperature.
