# Supplementary material for: Structural and Luminescence Behavior of Nanocrystalline Orthophosphate KMeY(PO4)2: Eu3+ (Me = Ca, Sr) Synthesized by Hydrothermal Method
Source: Materials (Basel). 2022 Mar 1;15(5):1850. doi: 10.3390/ma15051850 (PMC8911688; doi:10.3390/ma15051850)
Supplement: Supplementary file 1 [file materials-15-01850-s001.zip › materials-1595843-supplementary.pdf]

materials

MDPI

Supplementary Materials

Structural and Luminescence Behavior of Nanocrystalline Orthophosphate K*Me*Y(PO4)2: Eu^{3+} (*Me* = Ca, Sr) Synthesized by Hydrothermal Method

Aleksandra J. Pelczarska [1,*], Dagmara Stefańska [2,*], Adam Watras [2], Lucyna Macalik [2], Irena Szczygieł [1] and Jerzy Hanuza [2]

1 Department of Inorganic Chemistry, Faculty of Production Engineering, Wroclaw University of Economics and Business, Komandorska Street 118/120, 53-345 Wroclaw, Poland; irena.szczygiel@ue.wroc.pl

2 Institute of Low Temperature and Structure Research, Polish Academy of Science, Okolna Street 2, 50-422 Wroclaw, Poland; a.watras@intibs.pl (A.W.); l.macalik@intibs.pl (L.M.); j.hanuza@intibs.pl (J.H.)

* Correspondence: aleksandra.pelczarska@ue.wroc.pl (A.J.P.); d.stefanska@intibs.pl (D.S.)

Citation: Pelczarska, A.J.; Stefańska, D.; Watras, A.; Macalik, L.; Szczygieł, I.; Hanuza, J. Structural and Luminescence Behavior of Nanocrystalline Orthophosphate K*Me*Y(PO4)2: Eu^{3+} (*Me* = Ca, Sr) Synthesized by Hydrothermal Method. *Materials* **2022**, *15*, 1850. https://doi.org/10.3390/ma15051850

Academic Editor: Alexander N. Obraztsov

Received: 28 January 2022
Accepted: 25 February 2022
Published: 1 March 2022

Publisher's Note: MDPI stays neutral with regard to jurisdictional claims in published maps and institutional affiliations.

Copyright: © 2022 by the authors. Licensee MDPI, Basel, Switzerland. This article is an open access article distributed under the terms and conditions of the Creative Commons Attribution (CC BY) license (https://creativecommons.org/licenses/by/4.0/).

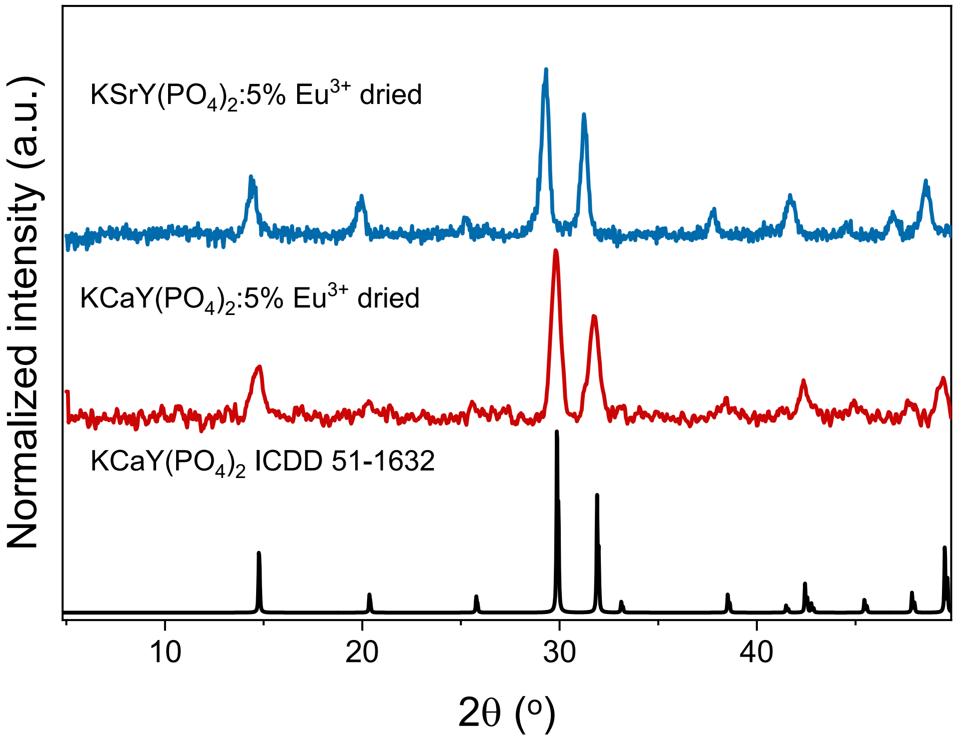

Figure S1. XRD pattern of obtained samples after hydrothermal process without annealing.

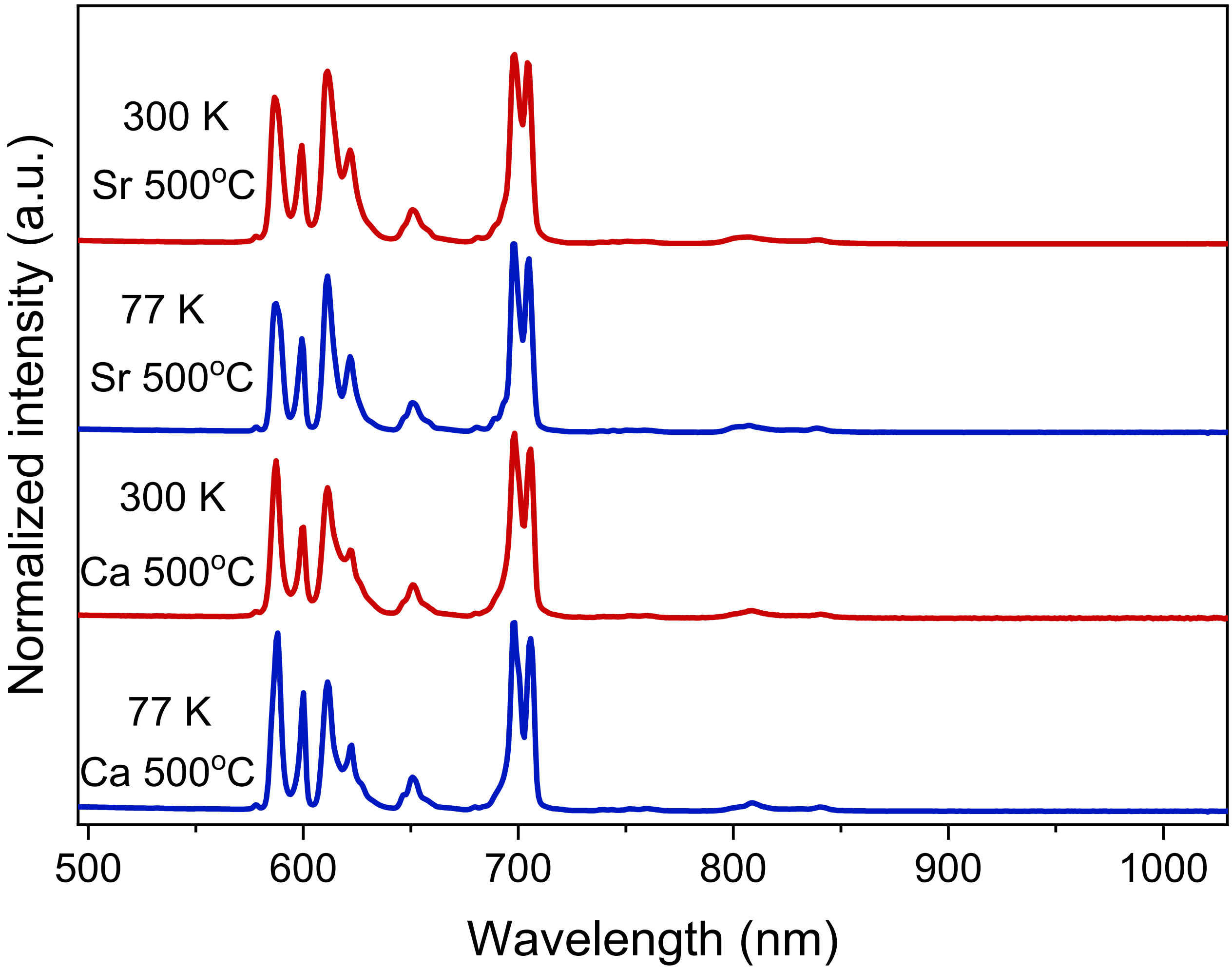

Figure S2. Emission spectra of representative samples $KMeY(PO_4)_2$ Me=Ca, Sr recorded at 77 and 300 K.

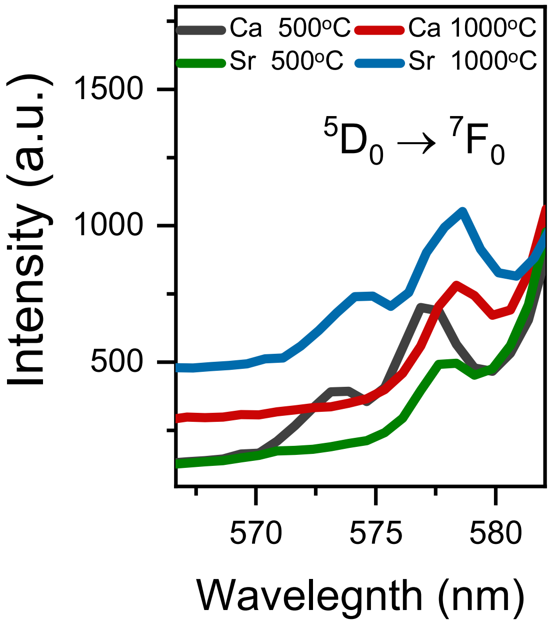

Figure S3. Magnification of the $^5D_0$-$^7F_0$ transition of investigated compounds.

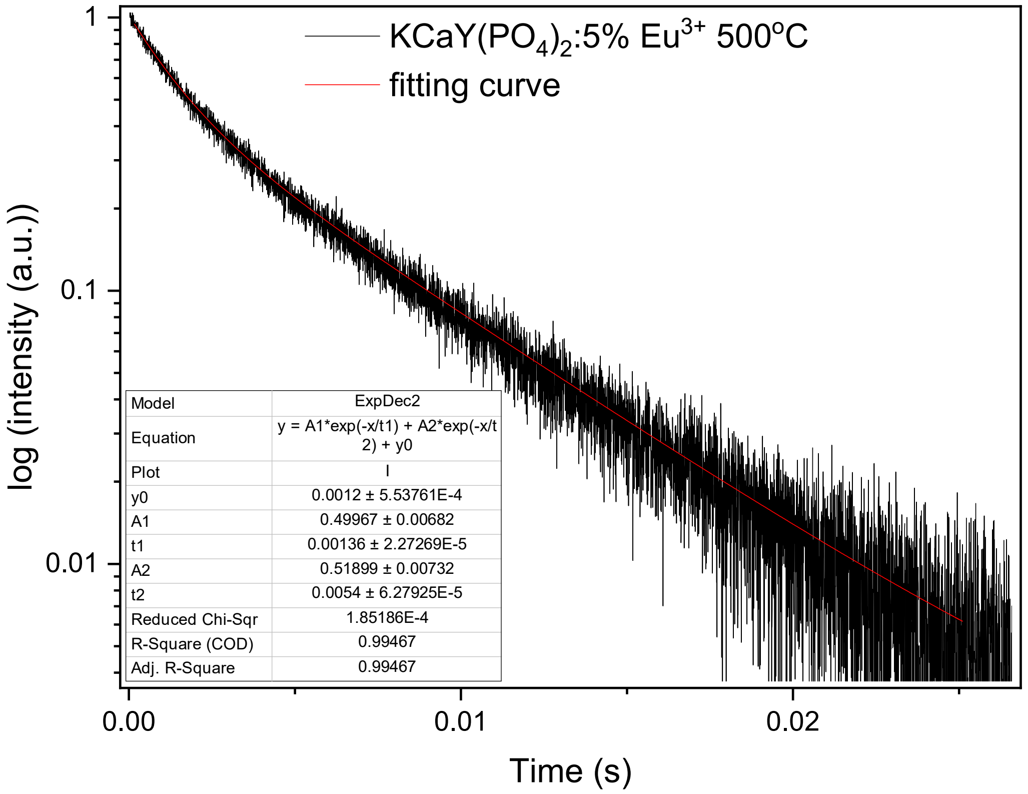

Figure S4. Emission decay curves of the $KCaY(PO_4)_2$: 5% Eu^{3+} samples annealed at 500°C and recorded at room temperature.

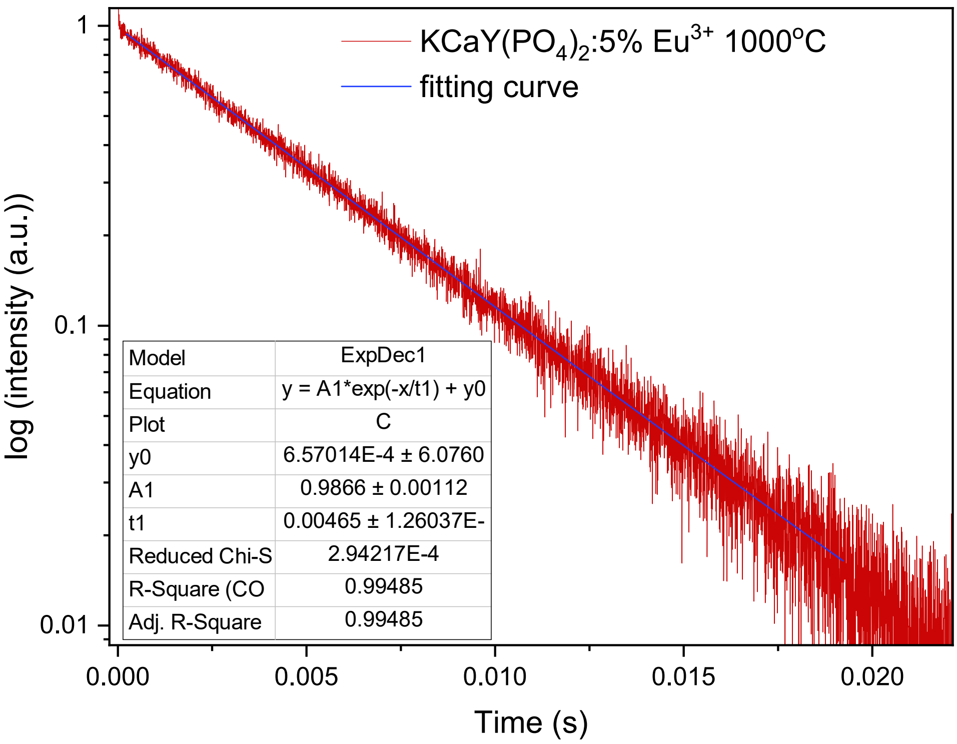

Figure S5. Emission decay curves of the $KCaY(PO_4)_2$: 5% Eu^{3+} samples annealed at 1000°C and recorded at room temperature.

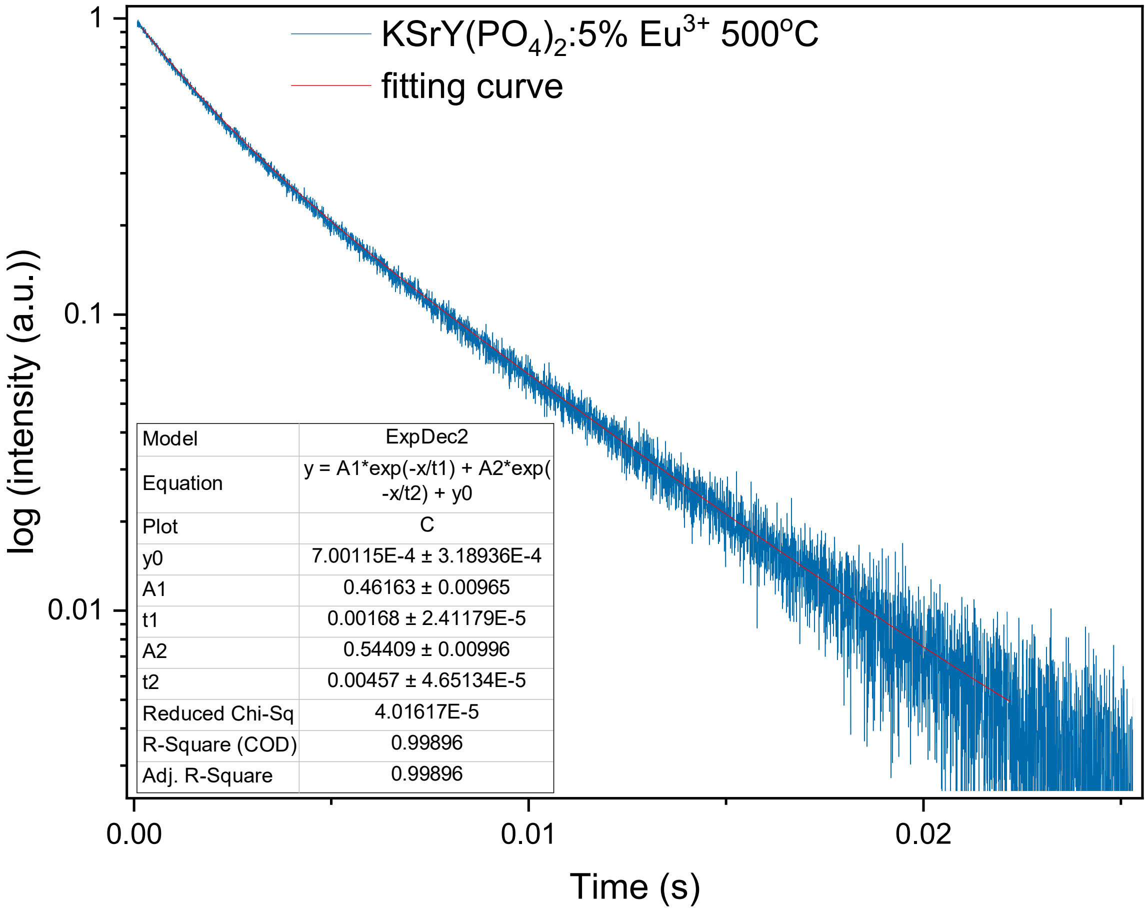

Figure S6. Emission decay curves of the $KSrY(PO_4)_2$: 5% Eu^{3+} samples annealed at 500°C and recorded at room temperature.

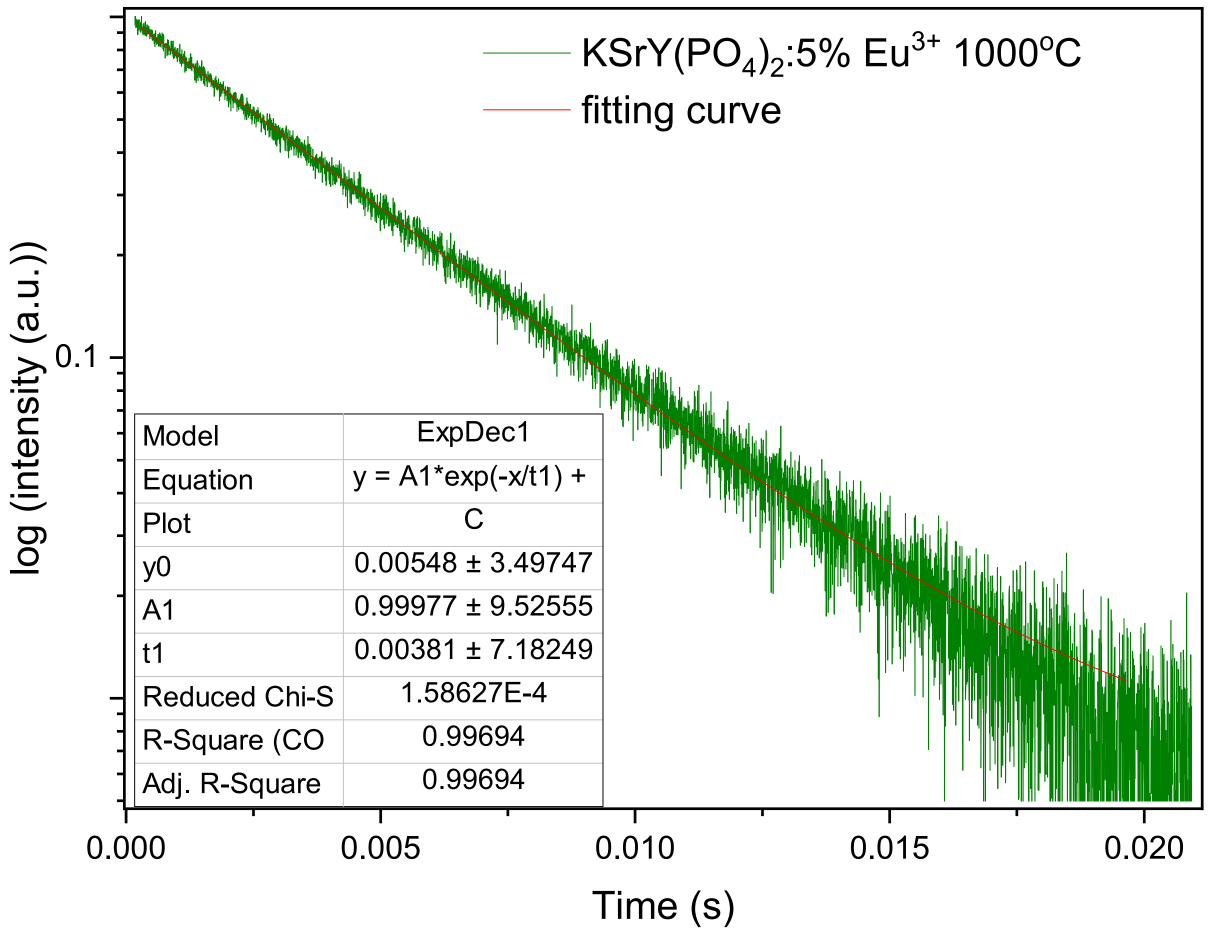

Figure S7. Emission decay curves of the $KSrY(PO_4)_2$: 5% Eu^{3+} samples annealed at 1000°C and recorded at room temperature.
